# Immunotherapeutic Strategies in the Management of Osteosarcoma

**DOI:** 10.26502/josm.511500076

**Published:** 2023-02-06

**Authors:** Rajiv Supra, Devendra K Agrawal

**Affiliations:** 1 College of Osteopathic Medicine, Touro University, Henderson, Nevada; 2 Department of Translational Research, Western University of Health Sciences, 309 E. Second Street, Pomona, California 91766-1854, USA

**Keywords:** Cancer immunotherapy, Cancer vaccine, Immune checkpoint inhibitors, Immunomodulation, Immunotherapy, Osteosarcoma, Tumor microenvironment, Vaccine therapy

## Abstract

Osteosarcoma (OS) is the most common primary malignant bone cancer with a high tendency for metastasis. Although treatment strategies involving surgery and chemotherapy have improved outcomes for patients with OS, the prognosis of recurrent OS is quite unsatisfactory. Primary reasons leading to mortality in OS patients are resistance to currently used therapies and the subsequent lung metastasis. Immunotherapy, however, has been shown to be a promising therapeutic strategy against OS. As research progresses, immunotherapy is gradually becoming irreplaceable. This article provides a critical evaluation of several therapeutic strategies for OS including immunomodulation, vaccine therapy, and immunologic checkpoint blockade.

## 1. Introduction

Osteosarcoma (OS), the most common primary bone tumor, has the highest incidence in adolescents and children with the second highest incidence in adults over the age of 60 [1]. The current clinical treatment involves surgery and chemotherapy that has shown to enhance the 5-year survival rate by >70% in patients with OS, however, it failed in OS patients with metastasis [2]. Additionally, it is more imperative than ever in those subjects with OS who have metastasis to other sites at the time of initial diagnosis [3]. Mainstream chemotherapeutic agents used in the treatment of OS include methotrexate, doxorubicin, ifosfamide, and cisplatin and have extensive side effect profiles. Side effects of these agents such as ototoxicity, mucositis, leukopenia, thrombocytopenia, and nephrotoxicity are problematic and challenging [4–6]. An appropriate command of the immune system using therapeutic agents is essential for tumor control and may provide better outcomes for patients with OS.

In this article, a critical discussion with the emphasis on immunotherapeutic strategies used for treating OS is presented. Strategies include immunomodulation, vaccines, blockage of immunologic checkpoints, and targeted therapy. Immunomodulation consists of activating components of the innate immune system, resulting in upregulating Natural Killer (NK) cells, monocytes, and macrophages, in an attempt to attack tumor cells [7]. Numerous clinical trials have been done on immunotherapies determining the efficacy and application towards treating OS [8,9]. The poor prognosis in patients with OS is a result of early hematogenous spread and resistance to therapy. Additionally, current treatments encounter bottlenecks in chemotherapy resistance and tumor immune escape, which are largely promoted by the microenvironment and tumor stem cells [10,11]. Novel immunotherapeutic strategies consisting of the application of tumor vaccines, genetically modified T cells, immune checkpoint inhibitors, and combination therapies can be used to mitigate treatment side effects, and improve the quality of life in patients with OS [12].

Thus, the focus of the article is on the immunotherapeutic strategies used in the management of OS highlighting the underlying mechanisms of action of immunotherapeutics with a view towards the future where cultivating the strength of these treatments could potentially lead towards a cure for OS.

## 2. Interaction of Tumor Cells with Immune cells in the Tumor Microenvironment

**Figure 1:**
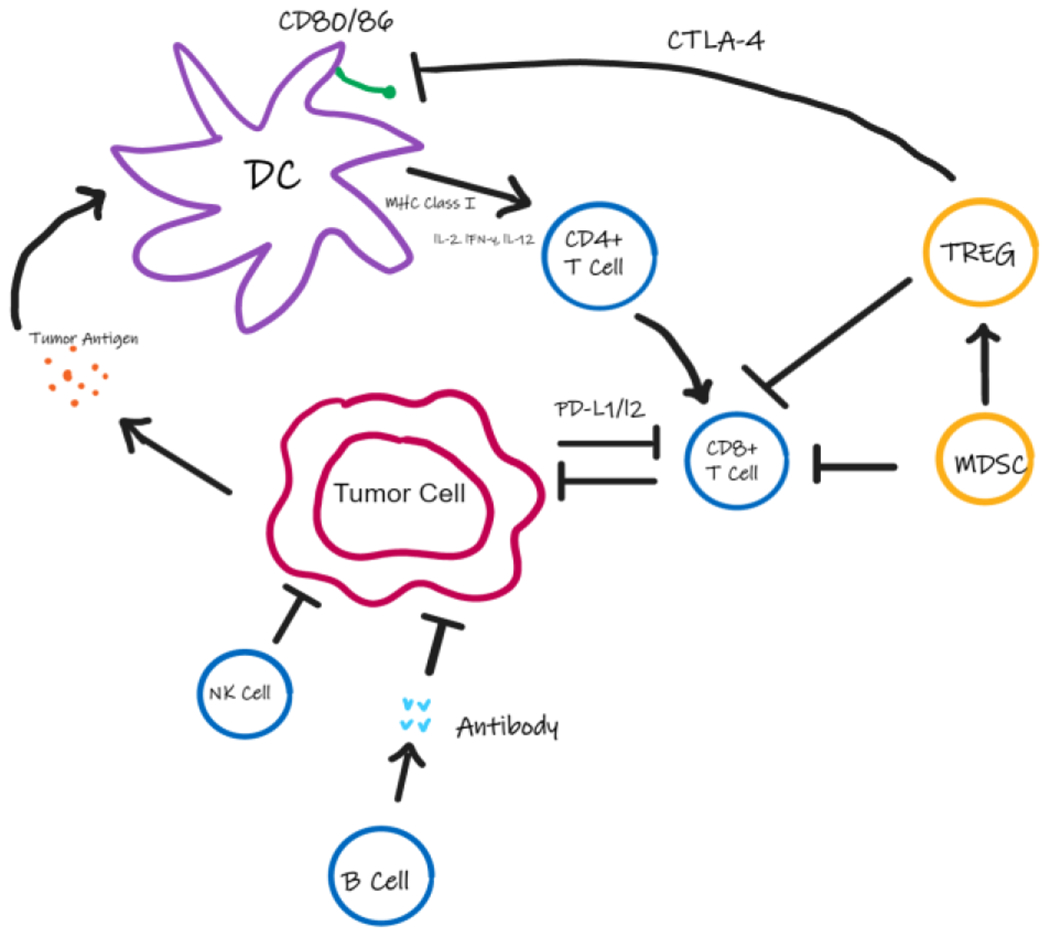
Interaction of Tumor cells with various immune cells. Tumor cells release tumor antigen which is taken up by the Dendritic Cells (DCs) to present to CD4+ T cells followed by the activation of CD8+ T cells. NK cells, B cells, T-Regulatory Cells (TREG) and Myeloid Suppressor Cells (MDSC) in the tumor microenvironment regulate the activation and function of DCs, CD8+ T cells and tumor cell. Expression of regulatory T cells and immune checkpoint proteins allow the tumor to evade the immune response.

The immune system consists of an intricate organization of cells that protect and fight against viruses and bacteria. The innate immune system consists of macrophages, NK cells, Dendritic Cells (DCs), neutrophils, eosinophils, and basophils. These cells are the primary defense against foreign pathogens. Mast cells and macrophages start the inflammatory response through various cytokines which interact with other immune cells. Antigen presenting cells, such as DCs, present foreign antigens to be recognized by adaptive immune cells. These adaptive immune cells consist of CD4+ T helper lymphocytes, B lymphocytes, and antigen-specific T lymphocytes. The adaptive and innate immune systems are interconnected and thus work together to eliminate foreign pathogens and remove damaged cells [13,14]. Normally, the adaptive and innate immune systems can detect tumor cells and destroy them through NK cells, secretion of interferon gamma (IFN-γ), and activating DCs. However, some cancerous cells evade this response and survive by various mechanisms like downregulating Major Histocompatibility Complex (MHC), recruiting regulatory T cells and myeloid suppressor cells, changing the tumor microenvironment, and upregulating inhibitory ligands and receptors on tumor cells and T cells, respectively [15,16] (Figure 1).

## 3. Macrophages in the Tumor Microenvironment and Novel Therapies

The immune microenvironment in osteosarcoma consists of both adaptive and innate immune cells, specifically T lymphocytes and macrophages [17]. These immune cells can be found in the adjacent lymphoid structures or in the core of the tumor [18]. OS cells are able to modulate both immune systems and therefore induce an immune-tolerant state which is conducive of metastasizing tumor cells [19]. OS patients that have higher immune scores with increased immune cells infiltrating the microenvironment have been documented to have a better prognosis than OS patients with lower immune scores [20]. Hence, the cross talk with tumor cells and the tumor immune microenvironment may be indispensable to further study and research for developing immunotherapies. Furthermore, macrophages are at a higher proportion of immune cells when compared to other immune cells in the tumor microenvironment of OS [21]. This reinforces the high plasticity of macrophages especially when presenting with inverse phenotypes and activating signals. For example, the pro-inflammatory phenotype of macrophages M1 and the anti-inflammatory phenotype M2, both serve different purposes depending on the immune response. When the immune system triggers tumor suppression, M1 macrophages serve as the major defense cells and cytokines such as IL-12, IL-6, and IL-1 are secreted [22–24]. In contrast, immune suppression is associated with the M2 macrophage response leading to tumor metastasis. Research has revealed that a higher proportion of tumor associated macrophages correlated with worse prognosis in most solid cancers and the M2 related cytokines showed increased events of lung metastasis in OS [25–27]. Interestingly, shifting the macrophage response from the M2 to M1 phenotype decreased incidence of lung metastasis in OS, further strengthening the role macrophages play in the tumor microenvironment [28]. However, controversy remains whether macrophages are pro-tumor or anti-tumor in the OS microenvironment. Previous research showed increased infiltration of macrophage in OS tumor cells, decreased rates of metastasis, and increased overall survival outcome, whereas M2 macrophage infiltration was associated with a poor prognosis [29,30]. Therefore, recent therapies have attempted to influence macrophage response within the tumor microenvironment. For example, All-Trans Retinoic Acid (ATRA) was proven to reduce pulmonary metastasis in OS through potentially indirectly deviating macrophages from M2 polarization [31,32]. Moreover, naturally occurring substances such as dihydroxycoumarins were also studied to induce macrophage polarization from M2 to M1 phenotype in OS treatments [33]. Dihydroxycoumadins inhibit the production of growth factors like TGF-β and cytokines such as IL-10. They also reduce the phosphorylation of STAT3 during M2 polarization, interfering with its activation [33,34]. Zoledronic acid, another potential therapeutic for OS, has been shown to interfere with M2 polarization causing macrophages in the tumor microenvironment to polarize back to the M1 phenotypes [35]. In a randomized study using zoledronate, however, the treatment using this agent did not result in improved outcomes in patients with OS [36]. Similarly, another study revealed zoledronate may induce bipotent macrophages by deleteriously polarizing to CD68+/CD163+ [37]. CD163 staining is associated with higher cMaf expression (a transcription factor associated with M2 macrophage polarization) while CD68 is stated to be an M1-polarized macrophage marker [38]. Therefore, the therapeutic potential of zoledronate still needs future research to elucidate whether it has a positive or negative effect on OS. Another novel immunomodulatory drug that increases inflammatory cytokines and triggers macrophage polarization is Mifamurtide [39]. Studies have demonstrated the potential of mifamurtide as an anti-tumor agent not only by restricting M2 initiation via decreased Akt phosphorylation and decreasing STAT3 levels, but also by shifting macrophage polarization to the intermediate phenotype M1-M2 [40]. Macrophage polarization has been a promising target for novel therapies in modulating the progression of OS.

## 4. Immune Checkpoint Inhibitors

Recent research has focused on immune checkpoint inhibitors and their association with the inhibition of tumor progression. Immune checkpoints enable immune tolerance against cancer in addition to preventing autoimmune disorders. Programmed death receptor-1 (PD-1) and CTLA-4 are the main inhibitory receptors on T cells and have been major candidates as targets for novel therapies against OS [41]. Normally, activated T cells express PD-1 on the surface and suppress the immune response through PD-L1, the ligand for PD-1, that is usually expressed on tumor tissues. Studies showed that anti-PD-1 therapy redirecting M2 macrophages to M1 resulted in regression of lung metastasis in a murine model of OS [42]. The efficacy of nivolumab in a murine model of OS was investigated and revealed nivolumab treated mice had significantly fewer rates of metastatic lung lesions, however, primary tumor volume and growth were not affected [43]. Another study revealed high levels of PD-L1 in OS patients and expression of PD-L1 was positively correlated with Tumor-Infiltrating Lymphocytes (TILs). The overall survival time of patients with low PD-L1 expression was 89 months and those with high levels of PD-L1 had a median survival duration of 28 months [44]. Hingorani et al. [45] revealed increased expression of immunosuppressive monocytes and increased CTLA-4 in T cells in patients with pediatric sarcoma. High levels of PD-1 expression was also noted on peripheral CD8+ and CD4+ T cells in OS patients and CD4+ T cells in patients with metastasis had significantly higher expression levels of PD-1 [46]. Moreover, combination therapy for OS has shown promising results using anti-CTLA-4 and anti-PD L1 antibodies which showed improved overall survival in a murine model with OS, whereas no benefits were noted when treated with anti-CTLA-4 antibody alone [47]. Although the therapeutic effects of combination immune checkpoint inhibitors have not been confirmed in clinical trials, there have been several reports that immunotherapies with nivolumab plus ipilimumab showed considerable tumor remission in metastatic OS patients [48,49]. Immunotherapies, however, do not come without adverse effects. Immune check point inhibitors like anti-PD-1 inhibitors are antigen agnostic which results in severe immune hyperactivation. This cytokine storm can result in encephalopathy, coagulation disorders, cerebrovascular events, and cardiovascular related events. The toxicities from these therapies can be neutralized by using steroids but impairs the effectiveness of the immunotherapies as a result. Studies showed concomitant medication of PD-1/PD-L1 immune check point therapies with prednisone can worsen the clinical outcomes in patients with OS [50,51]. Tocilizumab, an antibody that targets IL-6, was shown to successfully reduce adverse reactions from immunotherapies in a wide variety of models [52]. This reinforces the fact that antagonists of cytokine receptors in addition to immune check point inhibitor therapy can be a therapeutic option to help better manage OS in the future.

## 5. Adoptive T-Cell Therapy

Active and adoptive immunotherapies are used in the treatment of OS. Active immunotherapies using DCs, pulsed vaccines, and cytokines influence immune responses towards tumor cells. Adoptive immunotherapy involves administering ex vivo-expanded tumor specific cytotoxic immune cells [53]. This process includes harvesting autologous immune cells, expanding them using ex vivo cultures with modified T cell receptors or Chimeric Antigen Receptors (CAR) for adoptive therapy [54,55].

Tumor Associated Antigens (TAA) are recognized by engineered CAR T cells. The CAR includes an ectodomain consisting of a single chain variable fragment of an antibody which is able to identify TAAs. The endodomain is derived from CD3 zeta chain involved in intracellular signaling. The structure as a whole allows T cells to identify TAAs and result in tumor cytotoxicity in an MHC independent pathway [56]. HER2 has been shown to be expressed by OS cells in 40-60% of primary OS samples and the safety of HER2 CAR-T cells have been researched without dose limiting toxic reactions [57,58]. Interestingly, even with low levels of HER2 expression, CAR-T cells have been shown to effectively target HER2 which reinforces the potential CAR-Ts have to subvert cancerous cells. A recent study showed metastatic tumors that were not sensitive to chemotherapy were effectively depleted by CAR-Ts engineered on IL-11 receptor alpha chain and HER2 in OS murine models [57,59]. Additionally, CAR-T cells have been shown to inhibit Insulin-Like Growth Factor 1 (IGF-1) and receptor tyrosine and prolong the survival in OS mouse models [60]. The limitations using CAR-T include its side effects such as encephalopathies, end organ damage, and systemic inflammatory syndromes [61,62]. Future studies on CAR-T cell therapy will require addressing the drawbacks to this therapy and limiting the side effects in patients with OS.

Superior to CARs in targeting efficacy is the T Cell Receptors (TCRs) because they are able to interact with both surface antigenic peptides presented by HLA and tumors intracellularly [63]. This feature of TCRs allows for more advanced applications in solid tumors. Germline antigens from cancer cells are expressed in a reduced manner in tissues from different histologic origins. The germ cells lack MHC expression and are therefore protected from TCR T cell immunotherapy [64]. A specific cancer germline antigen, NY-ESO-1, is found in 31% of OS tumors and TCR therapy targeting this antigen is currently under investigation. Recent research revealed that the engineered CD8+ T cells can encode MHC-I restricted TCRs for therapy. Lu et al. [65] used CD4+ T cells transduced with MHC-II-restricted TCRs in patients with OS which resulted in objective partial responses in patients with metastatic lesions of the lung.

## 6. Cancer Vaccine Therapies

**Figure 2:**
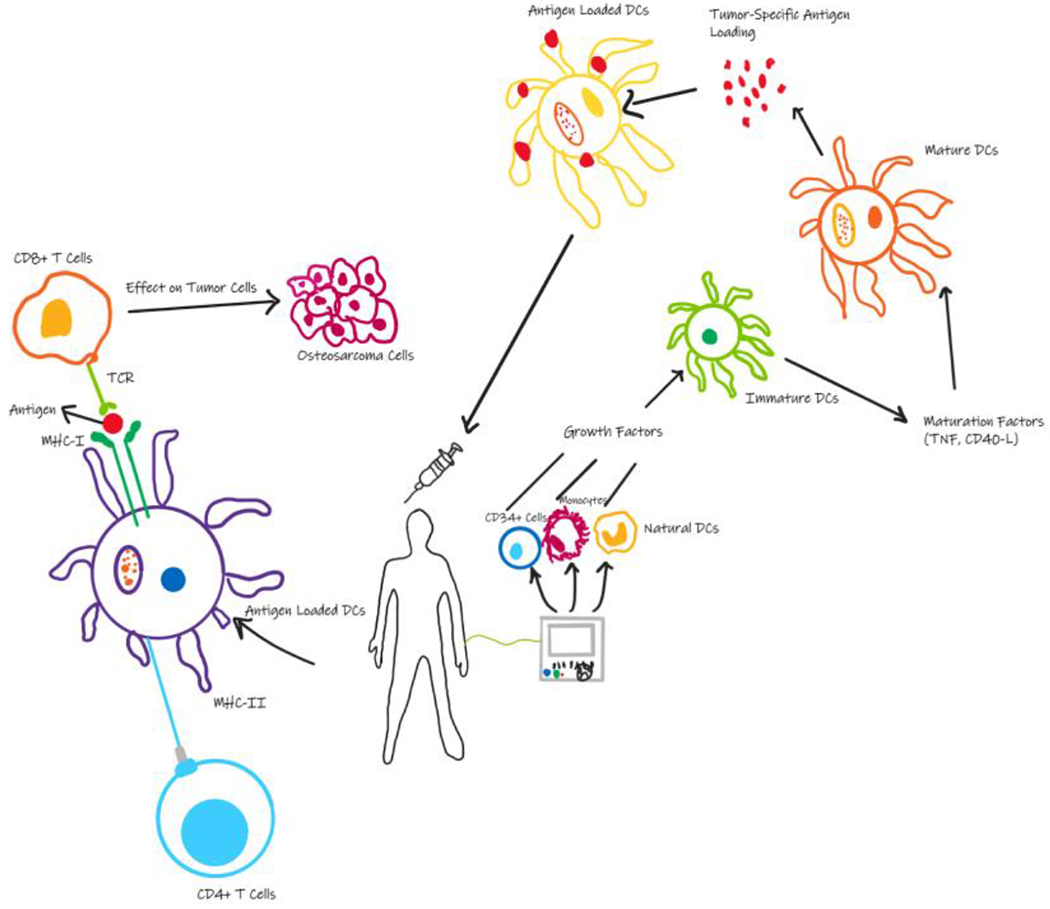
Process of engineering ex vivo DC vaccines. Progenitor cells are derived from patients and loaded with tumor antigens which subsequently active cytotoxic T cells to kill cancer cells.

Cancer vaccines have been studied and implemented to have anti-tumor effects by stimulating the immune response in patients. Peptides, DNA, RNA, and tumor antigens have been presented on cells to initiate the immune system [66]. Marcove et al. [67] initiated the use of autologous tumor lysates as vaccine therapies for cancer and resulted in increased survival rates in OS patients. Cancer vaccines have been classified into non-cell based, autologous, and tumor cell vaccines. Innate immune cells such as DCs, T cells, and macrophages are exploited using the immune cell vaccines. However, there are limitations to this mode of therapy mainly due to the immunosuppressive molecules within the tumor environment. The most widely used vaccination approach is through using DCs. These professional antigen presenting cells endocytose and present to naïve T cells that ultimately differentiate into tumor killing cells [68]. DC vaccines are able to reduce immunosuppression caused by cancerous cells and the latest FDA approved drug, ilixadencel, has shown great success [69]. The process of engineering DC vaccines first involves isolating peripheral mononuclear cells that are then pulsed with tumor antigen ex vivo. These cells are then injected back into the patient (Figure 2) [68]. DC vaccines can be classified based on the type of pulsed antigens for example, DCs can be co-cultured with tumor specific proteins, transfected with DNA coding for tumor antigens, or tumor lysates [69]. Studies have shown promising clinical responses with DCs pulsed with peptides from specific cancer germline antigens against OS [70]. Furthermore, combinational therapy using DCs and drugs targeting TGF-β suppresses the rates of metastasis through remodeling the tumor microenvironment [71]. CD14+ monocytes and CD34+ have been the main source of DCs in clinical trials in humans, however, Zhou et al. [72] affirmed that conventional type 1 dendritic cells can elicit tumor-specific T cell cytotoxicity in a murine OS model. This finding advances the horizon for using and developing DC vaccines for OS.

In addition to DCs, macrophages and γδ T cells have been used in tumor vaccines in the form of Chimeric Antigen Receptor Macrophages (CAR-Ms) and peptide-pulsed γδ T cells. CD8+ T cells are primed by the γδ T cells [73]. The γδ T cells have been reported to be superior to DC vaccines due to its HLA-independent manner in activating cytotoxic activity towards cancer cells [68]. Research revealed that γδ T cells can attack OS cells despite the limited susceptibility of cancer cells towards γδ T cell toxicity [74]. Rapamycin, an mTOR inhibitor can magnify the γδ T cell response by increasing tumor penetration [75]. Additionally, researchers recently manufactured novel CAR-Ms in order to phagocytose tumor cells, promote a pro-inflammatory tumor microenvironment through M2 to M1 polarization, and presenting antigens to naïve T cells subsequently influencing them to turn into cytotoxic T cells. An engineered anti-HER2 CAR-M induced a significant decrease in tumor burden and increased survival in murine models with metastatic lung cancer [76]. Polymer nanocarriers were fabricated in order to deliver genes encoding interferon-γ and CAR to macrophages in vivo and was able to code tumor specific CAR expressing macrophages in situ [77]. Collectively, these studies have potential to further our understanding of using γδ T cell and CAR- M vaccines against OS.

Autologous tumor vaccines are beneficial in that they bypass the culture ex vivo process and DC isolation and instead directly influence the DC response in vivo. As tumor cells are isolated from patients, they are expanded and irradiated before being re-infused [78]. A recent study revealed a combined autologous tumor vaccine with IL-2 together in canines with OS and resulted in prolonged survival compared to those that received amputation [79]. The safety and efficacy of single or combined autologous tumor cell vaccines has yet to be elucidated in human subjects. Viral and peptide vaccines have similar mechanisms of action by presenting the antigen to DCs in vivo. The tumor associated antigen Papilloma Binding Factor (PBF) derived peptide vaccine has long been researched for HLA-A2/A24+ OS patients. PBF is a transcription factor whose levels have been shown to reach up to 92% in OS patients. Peptides from PBF A24.2 are shown to activate cytotoxic T cells in OS patients positive with HLA-A24, and therefore can trigger the immune response to eliminate cancer cells [80]. A study was performed in creating a peptide tetramer targeting LSA and QVT peptides which showed toxicity against HLA-A11+PBF+ cells from OS [81]. Other vaccines such as those based on HER2 have been underappreciated by benefits seen in OS canine models. The recombinant listeria vaccine that targeted HER2 showed reduced levels of metastasis and improved prognosis when compared to the control group [82].

## 7. Cytokines

Studies revealed that cytokines can influence the immune response by interacting with immune therapeutic cells and other inflammatory cytokines such as IL-8, TNF-α which are correlated with the progression of OS [83]. Potentially the cytokine-based approaches have been the most widely used in immunotherapeutics and have been used for cancer therapy for over three decades. Their ability to activate T cells and modulate antigen presentation makes it a viable therapy option [53]. Macrophages release cytokines which then engage in then engage in the inflammatory response during cancerous states. IL-12, an immune-positive regulating cytokine can promote B and T cell differentiation, proliferation, and antibody formation. It has an integral role in reducing gastrointestinal bleeding caused by chemotherapy and improving patient tolerance to cancer radiotherapy. A study revealed that tumor cells from high grade OS patients were lysed by IL-15 induced NK cells [84]. In primary metastatic OS patients who were administered IL-2, a survival rate of more than 40% was documented at 3 years [85]. Limitations, however, exist with using cytokines due to their toxic side effect profile in patients with OS from administering a sufficient dose to activate the immune system [86].

## 8. Cytokines

Treatments for OS are transitioning from the standard therapies of chemotherapy and surgery towards more novel approaches such as immunotherapy. With improved understanding of the immune response to OS, many patients will benefit from immunotherapies for years to come. However, to date, new immunotherapeutic drugs have been limited in the use for OS. The main obstacle being limited T cell infiltration and immune hyperresponsiveness. Additionally, the tumor microenvironment makes these therapies difficult to penetrate into and kill cancer cells. Generally, immunotherapy shows promising data for treating OS, however further research is needed to determine more precise curative effects.

## Data Availability

Not applicable since the information is gathered from published articles.
